# Change of Editors

**Published:** 1857-06

**Authors:** 


### Change of Editors.

It may be a matter of surprise to our subscribers to see that the names of Professors McGugin and Allen no longer appear as editors of the Iowa *Medical Journal*. We do not doubt that it will be also a matter of regret that the *Journal* should have passed from their editorial charge into that of those who want the experience which has enabled our predecessors to make it so generally well received and highly prized by its readers. We partake in such a feeling, nor—could an arrangement have been made to retain the former editorial corps—would we have consented to assume the position we now occupy.

For ourselves we have only to say that it is with diffidence that we undertake the discharge of the duties of the post we have assumed. We shall attempt to be true to the trust and to maintain, as far as in us lies, the character which the *Journal* has acquired. We wish it to be a faithful exponent of the character and principles of legitimate medicine, as also to make it the organ of the Profession in the State, and of the contiguous portions of the adjoining States.

That we may not fail in our endeavors, it is necessary that we shall receive the aid of the profession. We would ask of you that you use the *Journal*, not as readers only of its pages, but as the organ through which you may yourselves be heard. Speak to each other through its pages. Contribute each your own share towards medical progress, by communicating the results of your observations and experience. Do not look to us to do all to make (what we wish to be considered as) *your* journal all that you wish it to be, for if you do we shall be able only to disappoint you. Let us rather be *your publishing* committee—your agents. If you will but do this, we shall be enabled to succeed in the little which will devolve upon us to do.

